# Psychosocial services provided by licensed cardiac rehabilitation programs

**DOI:** 10.3389/fresc.2023.1093086

**Published:** 2023-03-30

**Authors:** Montika Bush, Kelly R. Evenson, Aileen Aylward, Julianne M. Cyr, Anna Kucharska-Newton

**Affiliations:** ^1^ Department of Emergency Medicine, School of Medicine, University of North Carolina at Chapel Hill, Chapel Hill, NC, United States; ^2^ Department of Epidemiology, Gillings School of Global Public Health, University of North Carolina at Chapel Hill, Chapel Hill, NC, United States; ^3^ Department of Epidemiology College of Public Health, University of Kentucky, Lexington, KY, United States

**Keywords:** mental health assessments, barriers and facilitative factors, post-myocardial infarction care, patient beliefs and attitudes, cardiac rehabilitation

## Abstract

**Background:**

Professional health organizations recommend that outpatient cardiac rehabilitation programs include activities to optimize the physical, mental, and social well-being of patients. The study objectives were to describe among cardiac rehabilitation programs (1) mental health assessments performed; (2) psychosocial services offered; and (3) leadership's perception of barriers to psychosocial services offerings.

**Methods:**

A cross-sectional survey of North Carolina licensed outpatient cardiac rehabilitation programs on their 2018 services was conducted. Descriptive statistics were used to summarize survey responses. Thematic analysis of free text questions related to barriers to programmatic establishment or expansion of psychosocial services was performed by two team members until consensus was reached.

**Results:**

Sixty-eight programs (89%) responded to the survey. Forty-eight programs (70%) indicated offering psychosocial services; however, a majority (73%) of programs reported not directly billing for those services. At program enrollment, mental health was assessed in 94% of programs of which 92% repeated the assessment at discharge. Depression was assessed with the 9-item Patient Health Questionnaire by a majority (75%) of programs. Psychosocial services included individual counseling (59%), counseling referrals (49%), and educational classes (29%). Directors reported lack of internal resources (92%) and patient beliefs (45%) as the top barriers to including or expanding psychosocial services at their facilities.

**Conclusions:**

Cardiac rehabilitation programs routinely assess mental health but lack the resources to establish or expand psychosocial services. Interventions aimed at improving patient education and reducing stigma of mental health are important public health opportunities.

## Introduction

An estimated 805,000 myocardial infarction (MI) events occur in the U.S. each year (1). Most (85%) individuals survive until MI discharge but these patients are at increased risk for future cardiovascular events and death (1). In addition, mental health problems are common in this population. Depression occurs in an estimated 20% of patients surviving an MI (2–4) and is a recognized risk factor for poor cardiovascular outcomes (5). Depressed MI patients were 22% and 13% more likely than non-depressed patients to experience subsequent all-cause mortality or cardiovascular events, respectively (6). Comorbid depression and anxiety are also common among MI survivors (3, 7) and these condition increase their risk of mortality (8).

Comprehensive cardiac rehabilitation (CR) is a class I recommendation from the American Heart Association (AHA) to improve cardiovascular recovery and reduce mortality after MI (9–11). Within AHA's guidance for CR core components is the recommendation to include psychosocial management to reduce the impact of mental health problems on patient health outcomes (12). This recommendation appears alongside patient education for other well-known risk factors of post-MI morbidity and mortality such as uncontrolled hypertension and smoking. Exercise training, a core component of CR, has also been shown to independently improve symptoms of depression and anxiety (13). In addition to exercise, stress management is an evidence-based activity known to improve outcomes in CR participants when compared to programs that do not offer this service (14). Despite the importance of psychosocial services in the prevention of adverse health outcomes following MI, the details of how psychosocial services, including stress management, have been implemented and the barriers affecting implementation in CR are not well described in the literature.

In summarizing current CR practices, Hughes et al. identified the need for descriptive studies of behavioral health as a research priority (15). This current study addresses the need to describe the real-world implementation of psychosocial guidelines in CR programs and elucidates barriers to offering these programs despite AHA recommendation. For this study, we aimed to describe psychosocial and stress management services at outpatient CR facilities in the state of North Carolina (NC) and describe leadership's perspective on barriers to the use of these program offerings. To meet these objectives, we described CR program enrollment assessments, psychosocial and stress management service activities, and service personnel providing psychosocial and stress management services. Additionally, we gather qualitative data on leadership perceptions of barriers to CR psychosocial service and stress management initiation or expansion. Stratified summaries are presented since any potential future interventions might differ by level of current service offered. Comprehensive cardiac rehabilitation (CR) programs have the opportunity to identify patients at risk for mental health problems following an MI and to connect those patients with appropriate services. Our goal in describing assessments and services in NC CR is to call attention to the value of CR programs to provide this function and lay a foundation for future research and quality improvement for CR programs.

## Materials and methods

### Sampling frame and survey distribution

We conducted a cross-sectional survey of CR programs for the entire state of NC. Programs were identified from the NC Department of Health and Human Services Division of Health Service Regulation online directory of licensed facilities (accessed online Sep 2018). A unique link to the study questionnaire was sent *via* email to the program directors of 76 licensed CR facilities in NC to assess program offerings and obstacles to patient participation and program expansion. Directors were asked to provide details based upon what their program offered in calendar year 2018. Interviewees were offered a $25 incentive to complete the survey. We attempted to verify the email address of program directors before survey distribution; however, some messages were not delivered. Follow-up calls were made to CR facilities with rejected email addresses to obtain a valid email address. To promote survey completion, similar calls were made to programs which did not respond within two months of the initial survey distribution. We also encouraged program directors to designate a proxy to complete the survey if they were unavailable. The institutional Office of Human Research Ethics determined that this survey of program directors did not constitute human subjects research therefore did not require IRB approval.

### Survey design

The current study's survey expanded upon questions asked of NC CR programs in 1999 (16, 17) and 2004 (18). The original survey was designed to obtain descriptive information on CR facilities, personnel, services offered, and the patient population that is served by each program. An electronic version of the original paper survey was updated, pilot tested, revised, and distributed *via* email. New questions were developed to gain further details about psychosocial services currently provided and future intentions. Psychosocial and stress management services were ascertained separately; therefore, leadership could indicate whether they provide both, one, or neither of these services. New open-ended questions were asked to ascertain the types of program activities for psychosocial and stress management services separately (“Please provide a brief description of the program's psychosocial services.” and “Please provide a brief description of the program's stress management services.”). A single open-ended question was used to assess participants' beliefs regarding barriers to establishing or expanding program services (“In your opinion, what are the top 3 barriers to including or expanding psychosocial and/or stress management services within outpatient cardiac rehabilitation programs?”). New health questions assessed what instruments were used to collect health information (“Was the general health status of patients’ assessed with any of the following standardized instruments during 2018?”; “Was the mental health status of patients’ assessed with any of the following standardized instruments during 2018?”; “Was the social support of patients’ assessed with any of the following standardized instruments during 2018?”). Respondents could select multiple instruments if more than one was used during the calendar year. We also asked if instruments were administered at program enrollment, program conclusion and/or at periodic visit during cardiac rehabilitation. The final survey instrument contained sixty questions. Survey responses collected in a web-based survey tool, Qualtrics (Provo, UT), between February and June 2019 were exported and analyzed using SAS software version 9.4 (Cary, NC). The questionnaire was completed in 1 (SD:1.1) hour by 62% of respondents. The remaining respondents started and completed the survey on different days.

### Qualitative and quantitative data analysis

Two team members separately reviewed open-ended responses to the question regarding barriers to establishing or expanding program services. Initial code construction was completed using a consensus approach; team members proposed potential codes based on participant response patterns. This resulted in sixteen child codes. Team members then worked simultaneously to apply codes to each response, discussing and rectifying any disagreements. After the initial round of coding was complete, team members repeated this approach with the goal of synthesizing data into categories. This resulted in five themes relevant to program barriers. The approach of developing open-ended codes and further synthesizing codes into coherent categories for the purpose of elucidating themes was derived from Saldana's 2009 manual on qualitative coding (19).

For Tables 1 and 2, descriptive statistics were used to summarize survey responses for programs overall and by if psychosocial services were provided in that program. Standardized differences (20, 21) were computed to detect an imbalance in these non-randomized groups for quantitative survey questions. By convention, a 10% standardized difference between groups was considered meaningful (20). For Table 3, descriptive statistics were used to summarize survey responses by type of service. Psychosocial and stress management services could be provided independent of each other so are summarized separately. Descriptive statistics were used to summarize program barriers in Table 4 overall and by program psychosocial service providers excluding standardized differences since this was a summary of open-ended questions. The lead author had full access to all the data in the study and takes responsibility for its integrity and the data analysis. Anonymized data from this study are available from the corresponding author upon reasonable request.

**Table 1:** Characteristics of licensed certified outpatient cardiac rehabilitation programs.

Characteristic	Psychosocial Services		
	Non-Provider	Provider	Standardized
	*n* = 20	*n* = 48	Difference
**Overall Program Characteristics:**			
Mean (SD) Program Longevity, years	23.9 (10.45)	24.1 (10.52)	0.01
Did not respond [*n*(%)]	2 (10.0%)	1 (2.1%)	−0.34
Mean (SD) Patients Participating	231.2 (179.53)	270.1 (201.05)	0.20
Did not respond [*n*(%)]	0	5 (10.4%)	
Mean (SD) Weekdays Operational	4.1 ( 1.00)	4.0 ( 0.94)	−0.09
**Program Location Population Density**			
Rural (<250 population/square mile)	13 (65.0%)	23 (47.9%)	−0.35
**Outpatient program setting**			
Hospital	11 (55.0%)	31 (64.6%)	0.20
Other medical facility	4 (20.0%)	7 (14.6%)	−0.14
Rehabilitation center	4 (20.0%)	12 (25.0%)	0.12
Fitness facility, health club, or wellness center	4 (20.0%)	5 (10.4%)	−0.27
**Program Services:**			
**Other types of cardiac rehab supported**			
Maintenance (Phase III)	16 (80.0%)	31 (64.6%)	−0.35
Home-based, self-directed, or tele-monitored	0	1 (2.1%)	
Intensive cardiac rehab program	0	2 (4.2%)	
Stress Management Services Provided	17 (85.0%)	47 (97.9%)	0.47
**Program Personnel:**			
**Program Personnel, Median FTE (IQR)**			
Nurse (*n* = 66)	2.0 (1.6, 2.0)	2.0 (1.3, 2.5)	
Exercise physiologist (*n* = 65)	1.8 (1.0, 2.5)	1.5 (1.0, 3.0)	
Nutrition/dietitian (*n* = 53)	0.5 (0.3, 0.5)	0.5 (0.2, 0.5)	
Psychiatrist/Psychologist (*n* = 18)	0	0.3 (0.2, 0.3)	
Social worker (n = 16)	0.2 (0.2, 0.2)	0.3 (0.1, 0.7)	
Student in training (*n* = 10)	0.3 (0.3, 0.3)	1.0 (0.8, 1.0)	
Other: Respiratory Specialist (*n* = 19)	1.0 (0.8, 1.0)	1.0 (0.5, 1.0)	
Other: Administrative Staff/Management (*n* = 20)	1.0 (1.0, 1.0)	1.0 (1.0, 1.0)	
Other: Other Medical Professional (*n* = 6)	2.0 (2.0, 2.0)	1.0 (1.0, 2.0)	
Other: Stress Management Counselor (*n* = 2)	0.3 (0.3, 0.3)	0.5 (0.5, 0.5)	
Volunteers (*n* = 28)	0.2 (0.1, 2.0)	0.5 (0.3, 1.0)	
**Programs with Volunteers:**			
Programs Included	7 (35.0%)	21 (43.8%)	0.18
**Volunteer Activities**			
Administrative tasks	5 (71.4%)	16 (76.2%)	0.11
Social support	5 (71.4%)	16 (76.2%)	0.11
Cleaning equipment	6 (85.7%)	15 (71.4%)	−0.35
Checking patients in/out	2 (28.6%)	7 (33.3%)	0.10
Orientation assistant	2 (28.6%)	3 (14.3%)	−0.35
Blood pressure monitoring	1 (14.3%)	3 (14.3%)	
Other: Equipment Assistance	1 (14.3%)	2 (9.5%)	
Other: Patient Transportation	1 (14.3%)	1 (4.8%)	

Data presented as count (*n*) and proportion (%) of column population unless otherwise indicated. Highlighted cells have a meaningful standardized difference (≥10%).

FTE, Full-time equivalents; IQR, Interquartile Range; SD, Standard Deviation.

**Table 2:** Summary of psychosocial assessments performed at outpatient cardiac rehabilitation programs .

Characteristic	Psychosocial Services			Standardized
	Non-Provider	Provider	Overall	Difference
**Depression/Anxiety**				
Programs Included (denominator)	19	45	64	−0.05
**Which instrument used?**				
Beck Depression Inventory - II	1 (5.3%)	9 (20.0%)	10 (15.6%)	0.45
Center for Epidemiologic Studies-Depression Scale	2 (10.5%)	3 (6.7%)	5 (7.8%)	−0.14
Hospital Anxiety and Depression Scale	2 (10.5%)	0	2 (3.1%)	.
Patient Health Questionnaire-9	14 (73.7%)	34 (75.6%)	48 (75.0%)	0.04
Other: Generalized Anxiety Disorder (GAD-7)	1 (5.3%)	3 (6.7%)	4 (6.3%)	0.06
Other: Zung Self-Rating Depression Scale (SDS)	1 (5.3%)	1 (2.2%)	2 (3.1%)	−0.16
Other Instrument	0	2 (4.4%)	2 (3.1%)	.
Multiple instruments used?	2 (10.5%)	7 (15.6%)	9 (14.1%)	0.15
**Timing of Assessment**				
At program enrollment only	1 (5.3%)	3 (6.7%)	4 (6.3%)	0.06
At program enrollment and conclusion	18 (94.7%)	41 (91.1%)	59 (92.2%)	−0.14
Any periodic assessments	3 (15.8%)	10 (22.2%)	13 (20.3%)	0.16
**Health-Related Quality of Life**				
Programs Included (denominator)	17	43	60	0.14
**Which instrument used?**				
12-Item Short Form Health Survey (SF-12)	0	1 (2.3%)	1 (1.7%)	.
36-Item Short Form Health Survey (SF-36)	0	7 (16.3%)	7 (11.7%)	.
Dartmouth Cooperative Functional Assessment Charts	5 (29.4%)	6 (14.0%)	11 (18.3%)	−0.38
Ferrans and Powers Quality of Life Index - Cardiac	12 (70.6%)	31 (72.1%)	43 (71.7%)	0.03
MacNew Health-related Quality of Life	0	1 (2.3%)	1 (1.7%)	.
Psychosocial Risk Factor Survey (Delta Psychology Center)	0	2 (4.7%)	2 (3.3%)	.
Other: Herridge Cardiopulmonary Questionnaire	1 (5.9%)	1 (2.3%)	2 (3.3%)	−0.18
Multiple instruments used?	1 (5.9%)	6 (14.0%)	7 (11.7%)	0.27
**Timing of Assessment**				
At program enrollment only	0	2 (4.7%)	2 (3.3%)	.
At program enrollment and conclusion	17	40 (93.0%)	57 (95.0%)	−0.39
Any periodic assessments	2 (11.8%)	11 (25.6%)	13 (21.7%)	0.36
**Social Support**				
Programs Included (denominator)	5	6	11	−0.41
**Which instrument used?**				
PROMIS	1 (20.0%)	1 (16.7%)	2 (18.2%)	−0.09
ENRICHD Social Support Index (ESSI)	0	1 (16.7%)	1 (9.1%)	.
Interview/Other Instrument	4 (80.0%)	4 (66.7%)	8 (72.7%)	−0.30
**Timing of Assessment**				
At program enrollment only	0	3 (50.0%)	3 (27.3%)	.
At program enrollment and conclusion	5	3 (50.0%)	8 (72.7%)	−1.41
Any periodic assessments	2 (40.0%)	2 (33.3%)	4 (36.4%)	−0.14

Highlighted cells have a meaningful standardized difference (≥10%). Not all programs administered each type of assessment.

PROMIS, Patient-Reported Outcomes Measurement Information System.

**Table 3:** summary of mental health related services at outpatient cardiac rehabilitation programs.

Characteristic	Stress Management Services	Psychosocial Services
	*n* = 64	*n* = 48
Mean (SD) Patients Participating	161.6 (144.76)	134.8 (163.04)
Did not respond	13	9
Mean (SD) % of Patients Participating	64.6 (31.50)	52.1 (36.67)
Did not respond	15	11
**Types Of Activities**		
Educational Classes	23 (63.9%)	12 (29.3%)
Individual Counseling	8 (22.2%)	24 (58.5%)
Referral to Counseling or Other Services	5 (13.9%)	20 (48.8%)
Skills and Technique Practice	7 (19.4%)	0
Educational Materials Provided	4 (11.1%)	0
Other	5 (13.9%)	5 (12.2%)
Did not respond	28	7
**Typical Provider of Service**		
Nurse	28 (44.4%)	15 (31.3%)
Exercise physiologist	12 (19.0%)	1 (2.1%)
Psychiatrist/Psychologist	12 (19.0%)	17 (35.4%)
Social worker	7 (11.1%)	11 (22.9%)
Other Role	4 (6.3%)	4 (8.3%)
**Insurance Billed for Service**		
Always	2 (3.2%)	5 (10.4%)
Most of the time	0	1 (2.1%)
Sometimes	5 (7.9%)	2 (4.2%)
Never	51 (81.0%)	35 (72.9%)
Unknown	5 (7.9%)	5 (10.4%)
**Service Availability**		
1 Day per week	29 (48.3%)	22 (50.0%)
2–3 Days per week	24 (40.0%)	14 (31.8%)
4–5 Days per week	7 (11.7%)	8 (18.2%)
Weekday (after 5 pm)	2 (3.3%)	1 (2.3%)
Did not respond	4	4

Data presented as count(n) and proportion (%) of column population unless otherwise indicated. SD, Standard Deviation.

**Table 4:** Perceived barriers to including or expanding psychosocial or stress management services in outpatient cardiac rehabilitation program.

Barriers	Psychosocial Services		
	Non-Provider	Provider	Overall
	*n* = 20	*n* = 40	*n* = 60
**Current service practices**			
External Reimbursement	1 (5.0%)	7 (17.5%)	8 (13.3%)
Bundled Services	2 (10.0%)	1 (2.5%)	3 (5.0%)
Referral Resources	2 (10.0%)	0	2 (3.3%)
**Organizational beliefs**			
Organizational Knowledge	1 (5.0%)	3 (7.5%)	4 (6.7%)
Organizational Support	1 (5.0%)	5 (12.5%)	6 (10.0%)
**Internal resources**			
Organization - Expense	6 (30.0%)	22 (55.0%)	28 (46.7%)
Organization - Staffing	10 (50.0%)	15 (37.5%)	25 (41.7%)
Environment - Time	8 (40.0%)	12 (30.0%)	20 (33.3%)
Organization - Ability	4 (20.0%)	7 (17.5%)	11 (18.3%)
Environment - Space	2 (10.0%)	7 (17.5%)	9 (15.0%)
**Patient resources**			
Time/Money	1 (5.0%)	2 (5.0%)	3 (5.0%)
Health/Support	0	2 (5.0%)	2 (3.3%)
**Patient beliefs**			
Low Interest	4 (20.0%)	11 (27.5%)	15 (25.0%)
Reticence	2 (10.0%)	10 (25.0%)	12 (20.0%)
Other	0	2 (5.0%)	2 (3.3%)

Each program could provide up to 3 responses. Eight programs did not respond to these questions.

## Results

Sixty-eight programs (89%) across North Carolina participated in this cross-sectional survey. A majority of programs (69%) offered maintenance (i.e., phase III) CR but very few offered an intensive CR program (3%) or a home-based CR program (1%) (Table 1). Most programs (70%) reported offering psychosocial services and almost all (94%) programs reported offering stress management services (Table 1). Programs which offered psychosocial services had on average slightly higher patient populations during the year 2018 (Mean: 270, SD: 201) than programs not offering these services (Mean: 231, SD: 180). The qualifying event for 75% of patients attending CR sessions at responding programs was either MI, coronary artery bypass grafting, angioplasty/stent placement. There were only minor differences in demographic characteristics and qualifying events between programs that did and did not offer psychosocial services. Overall, the typical (median proportion ≥60%) characteristics of program participants included White race, male sex, at least 65 years old, English speaking, and Medicare insured.

In line with AHA and American Association of Cardiovascular and Pulmonary Rehabilitation (AACVPR) (12) guidelines, psychosocial assessments were completed at CR program enrollment and at additional times depending upon the type of assessment. Depression/anxiety specific instruments were used in 64 (94%) programs and health-related quality of life (QoL) instruments were used in 60 (88%) programs (Table 2). QoL instruments assessed psychological symptoms as well as other factors related to quality of life such as physical functioning. Eighty-two percent of CR programs used both depression/anxiety and QoL instruments in 2018. Social support was infrequently (16%) assessed independently of QoL. There was no consensus method of assessing social support from study participants.

The nine-item Patient Health Questionnaire (PHQ-9) (22), the most common depression assessment instrument, was used by 75% of programs (Table 2). The Beck Depression Inventory (23) was also used by 20% of programs providing psychosocial services. Approximately 13% of programs reported using more than one depression/anxiety assessment instrument during the study period. The seven-item Generalized Anxiety Disorder (24) assessment and PHQ-9 were administered by 4 programs. Other programs administered the PHQ-9 and either the Center for Epidemiologic Studies-Depression Scale (25) (*n* = 2) or Beck Depression Inventory (*n* = 2). All programs that performed a depression/anxiety assessment did so at program enrollment. Over 90% of these programs also assessed depression/anxiety at program conclusion. Between enrollment and program conclusion, 20% of programs periodically reassessed depression/anxiety.

The Ferrans and Powers Quality of Life Index—Cardiac questionnaire (26) was the most frequently administered (72%) health-related QoL assessment (Table 2). The Dartmouth Cooperative Function Assessment (27, 28), the second most popular of these types of assessments, was administered by a greater proportion of programs not providing psychosocial services than programs providing these services. Short Form Health Surveys (29, 30) (both 36- and 12-item) were used by psychosocial service providing programs but not by non-providing programs. Health-related QoL was assessed at program enrollment and conclusion by 95% of programs that completed this type of assessment. A larger proportion of psychosocial service providing programs than non-providing programs completed additional periodic QoL assessments between enrollment and program conclusion.

Programs that indicated providing psychosocial services and stress management services were also asked to provide details of activities associated with these services (Table 3). A majority (64%) indicated providing educational stress management classes. Several programs also provided individual counseling (22%) and/or skills/technique practice (19%) as stress management activities. A smaller proportion (14%) provided a referral for other stress management services. On average, program leadership reported that 65% of CR participants attended at least one stress management session when offered. Nurses were the most common healthcare provider utilized to deliver stress management services, 44% of programs. Stress management services were also provided by either an exercise physiologist, psychiatrist, and/or psychologist in 19% of programs. Almost half (48%) of programs provided stress management 1 day a week and few (12%) offered these services 4–5 days a week. While these services were typically provided before 5pm, two programs provided these services after 5 pm.

Individual counseling (59%) and referral to counseling (49%) were the top psychosocial services provided by responding programs. Psychosocial educational classes were also provided by 29% of programs. On average, psychosocial services were utilized by 52% of CR participants. The top 3 professions providing psychosocial services were physiologists/psychiatrists (35%), nurses (31%), and social workers (23%). Similar to stress management services, psychosocial services were offered once a week by 50% of programs; however, more programs (18%) offered psychosocial services 4–5 days a week. Only 1 program reported providing psychosocial services after 5 pm.

Sixty programs (88%) responded with their top three barriers to expansion of psychosocial or stress management services provided in outpatient CR programs (Table 4). Free-text responses were grouped into 5 main categories (Current service practices, Organizational beliefs, Internal resources, Patient resources, and Patient beliefs) with multiple subcategories. The top 3 barriers identified by program directors were lack of internal resources related to organizational expense (47%), organizational staffing (42%), and environmental time (33%). These categories were derived from survey responses like “budget constraints/costs”, “staffing resources/availability of resources” and “time constraints/schedule” respectively. Patient beliefs were identified by 45% of programs as a barrier. Specifically, both low patient interest and patient reticence were reported by at least 20% of respondents. Survey responses used to derive these categories included “patient interest/patient participation” and “patient unwilling/fear.”

## Discussion

Since mental health assessment is one of the recommended core components of CR, CR programs provide an opportunity to identify participants with psychosocial risk factors for poor cardiovascular outcomes. CR programs not only have the capacity to identify participants who are at high risk of psychological distress at clinical diagnosis thresholds but also those patients with no/low psychological symptom burden initially who may progress to clinical diagnoses. Since psychological symptom burden changes over time, CR programs afford the exceptional opportunity to monitor symptom persistence within the early recovery period after an acute event.

All programs in this study assessed for psychological symptom burden at program enrollment using depression screening instruments or as a component of a health-related quality of life assessment. NC CR programs also have excellent surveillance of changes in patients' symptom burden with approximately 95% of programs administering these assessments more than once. The high assessment rate at program entry and exit is consistent with what has been reported in other high-income countries (31, 32) and represents a marked increase in assessment rates of 29% to 68% observed across the globe between 1995 and 2010 (33, 34). Similar to Australian practice (32), validated instruments were used to assess depression and/or anxiety in NC CR programs but it was unlikely that validated instruments were used to assess social support. Social support was also less likely to be assessed in NC CR programs than Australian CR programs (16% vs. 80%) (32).

The type of healthcare system will influence the type of subsequent psychosocial health services. Results from this study and Canada indicate that 50%-60% of programs offer psychological counseling (31) while psychosocial follow-up across a survey of European countries was over 60% (34). However, not all respondents to our survey indicated offering psychosocial services and those that did also indicated referral to another entity for counseling. Cahill et al. summarized the effects of depression screening on depression outcomes (33), however, outcomes were not the focus of this research project since we did not assess patients directly. Future research could investigate the influence of models of mental health service on physical and mental health outcomes including adherence to CR participation and of other lifestyle changes.

Psychosocial services are an essential component of CR programs; however, this study identified barriers to implementing these services in practice. Insurance coverage constraints fuel financial barriers to psychosocial services within CR programs. For example, Medicare coverage focuses on medically supervised exercise training with ECG monitoring, as necessary, for 2 to 3 sessions per week up to 12 to 18 weeks without the need for special permission (35). Medicare policy updates have required the inclusion of education and counseling without increasing the number of covered sessions resulting in the majority of NC CR programs providing such services without insurance reimbursement (Table 3). Financial barriers to CR implementation were not just a problem in this study population but were prevalent in other countries (32, 36, 37).

Outside of changes to the payment structure for CR programs, additional effort is necessary to remove the perceived stigma of addressing mental health among patients. To reap the benefit of psychosocial services, patients need a greater awareness of the connection between mental and physical health and of the benefits of addressing both aspects of their health following a cardiac event. Patients do not feel that they need CR in general or psychosocial services in particular (38, 39). Given the low numbers of eligible patients attending CR, overall patient participation in CR programs needs to be increased to maximize benefits of psychosocial services in cardiovascular disease patients (40–42).

Comprehensive cardiac rehabilitation recommendations (12) address multiple evidence-based risk factors for poor health outcomes after an acute coronary event. Although mental health status is an established risk factor for poor physical health outcomes in heart disease patients, there is still controversy on if mental health assessments should occur in these patients (43, 44). Resistance to implementing psychological screening in outpatient practice exists despite recommendation for outpatient depression screening for the general public and patients with cardiovascular disease specifically (45). Treatment initiation requires evaluation. CR programs are one avenue where evaluation can routinely occur.

This study described the psychosocial aspects from a broader survey designed to summarize services offered by CR programs in North Carolina (46). The strength of this study is that it is one of the first statewide descriptions of CR program services and referral processes (33). Considering the limited research in this area, this study provides a guide for other states to identify state and local level practices for advancement and quality improvement of CR programs as well as resource needs to improve mental and physical collaborative care for patients after a cardiac event. The potential for information bias is a limitation of this study. However, programs that participate in the AACVPR registry, other quality improvement programs, or have organizational requirements for data such as those collected in this study will have access to more accurate information than programs that do not participate in those activities. In addition, most questions were straightforward and familiar to those respondents who participated in the two previous surveys of NC CR programs.

Despite its limitations, this statewide study is important because by highlighting that the mental health of CR patients will be routinely assessed it illustrates how CR can play a role in optimal health care of patients. Statewide, CR programs were compliant with psychosocial assessment and regardless of restricted funding, a majority of CR programs provide at least basic psychosocial services. Psychosocial services and education are a critical component of post-acute care that needs additional support for improvement in the comprehensive care of patients recovering from cardiac events. Future research could include investigate implementation of different payment models to overcome financial barriers to program enhancement. Literature suggests there are numerous barriers to integrating psychosocial services into primary care type settings (47, 48); therefore, implementation research into facilitators for psychosocial follow-up among the sizeable number of programs not currently providing psychosocial services is also needed. In addition, more research is needed on the assessment and mental health status of CR eligible patients that do not attend at least one CR session since only 30% to 50% of eligible patients attend at least one session of CR (40, 42).

## Data Availability

Data generated for this study are available upon reasonable request to the corresponding author in a de-identified format after the requestor completes our data use agreement.
